# Will COVID‐19‐related economic worries superimpose health worries, reducing nonpharmaceutical intervention acceptance in Germany? A prospective pre‐registered study

**DOI:** 10.1002/ijop.12753

**Published:** 2021-03-16

**Authors:** Tom Rosman, Martin Kerwer, Holger Steinmetz, Anita Chasiotis, Oliver Wedderhoff, Cornelia Betsch, Michael Bosnjak

**Affiliations:** ^1^ Leibniz Institute for Psychology (ZPID) Universitätsring 15 Trier Germany; ^2^ University of Erfurt Erfurt Germany

**Keywords:** COVID‐19, Nonpharmaceutical interventions, Social distancing, Virus‐related worries, Economic worries

## Abstract

Nonpharmaceutical interventions (NPI) such as stay‐at‐home orders aim at curbing the spread of the novel coronavirus, SARS‐COV‐2. In March 2020, a large proportion of the German population supported such interventions. In this article, we analyse whether the support for NPI dwindle with economic worries superimposing virus‐related worries in the months to follow. We test seven pre‐registered^1^ hypotheses using data from the German COSMO survey (Betsch, Wieler, Habersaat, et al. 2020), which regularly monitors behavioural and psychological factors related to the pandemic. The present article covers the period from March 24, 2020 to July 7, 2020 (*N*_total_ = 13,094), and, in addition, includes a validation study providing evidence for the reliability and validity of the corresponding COSMO measures (*N* = 612). Results revealed that virus‐related worries decreased over time, whereas economic worries remained largely constant. Moreover, the acceptance of NPIs considerably decreased over time. Virus‐related worries were positively associated with acceptance of NPIs, whereas this relationship was negative regarding economic worries (albeit smaller and less consistent). Unexpectedly, no interactions between virus‐related worries and economic worries were found. We conclude that individual differences in virus‐related and economic threat perceptions related to COVID‐19 play an important role in the acceptance of NPIs.

By the end of 2019, a novel coronavirus, SARS‐CoV‐2, emerged. Despite initial containment efforts, the virus quickly became pandemic, leading to hundreds of thousands of deaths related to the corresponding disease, COVID‐19. To limit the spread of the virus, nonpharmaceutical interventions (NPI) such as stay‐at‐home orders were introduced by a large number of countries. However, such behavioural measures are only effective to the extent that individuals adhere to them. As NPIs may have side effects—most notably for economic factors—monitoring subjective evaluation of NPIs, including their expected negative effects, is crucial. For this reason, Betsch and colleagues published a monitoring instrument on individual behaviour during the pandemic, COVID‐19 Snapshot Monitoring (COSMO Germany; Betsch, Wieler, Habersaat, et al., 2020). COSMO includes “variables that are critical for behaviour change in the population to avoid transmission of COVID‐19, including risk perceptions, trust, use of information sources, knowledge as well as barriers and drivers to recommended behaviours” (WHO Regional Office for Europe, 2020, p. 9). Together with the World Health Organization (WHO) Europe, the instrument was later adapted for international use.

Since knowledge on the psychological factors that affect the acceptance of NPIs is still rather scarce, the present article investigates, using the COSMO Germany data, the interplay between virus‐related health worries and economic worries on the acceptance of state‐imposed NPIs in Germany. Drawing on well‐established psychological theory (e.g., Tversky & Kahneman, 1973), we investigate whether, over the period from March 24, 2020 to July 7, 2020, virus‐related health worries decreased, whereas economic worries increased, leading to a reduction in the acceptance of NPIs. As regularly administering the COSMO instrument demands a particularly short survey, it is largely based on single item measures. Hence, we further present a validation study testing the reliability and construct validity of key COSMO measures.

The structure of this article is as follows: after deriving a set of seven pre‐registered hypotheses, we report results from the item validation study (Study 1), in which we surveyed a broad, quota‐based sample (*N* = 612) to gain data on the factorial validity and reliability of the COSMO measures. In Study 2, we analyse our main research question, namely the interplay between virus‐related health worries and economic worries on the acceptance of NPIs, using data from 13 COSMO Germany waves (*N* = 13,094).

## BACKGROUND AND HYPOTHESES

From March 2020 on, prohibition of gatherings, school closings, and even general curfews were enforced by a vast number of European countries. Such measures have been shown to be effective in curbing the spread of a pandemic because they reduce the likelihood that individuals catch the virus and subsequently infect other persons (Anderson et al., 2020; Hsiang et al., 2020). NPIs, however, require a close monitoring of individual compliance since they entail considerable limitations to individual freedom, thus bearing potential for reactance and opposition on the part of the population.

Nevertheless, at the beginning of the pandemic, a large proportion of the German population complied with, supported, and even requested the implementation of these measures (Betsch, Korn, Felgendreff, et al., 2020; Betsch, Wieler, Bosnjak, et al., 2020; Rieger & Wang, 2020). From a psychological standpoint, this is hardly surprising. In fact, research on risk perception shows that humans tend to overestimate unknown, extraordinary, and emotionally salient risks, whereas they underestimate more common everyday risks (e.g., Fischhoff et al., 1978; Loewenstein et al., 2001). This may be because of a phenomenon called the *availability heuristic* (Tversky & Kahneman, 1973). In fact, already in 1973, Tversky and Kahnemann argued that judging the exact likelihood of certain events (such as a COVID‐19 infection) is difficult, and, therefore, “people employ a limited number of heuristics which reduce these judgments to simpler ones” (Tversky & Kahneman, 1973, p. 207). The availability heuristic is one such mental shortcut, and suggests, according to Tversky and Kahneman (1973), that individuals infer an event's likelihood as higher when information relating to that event is readily available (e.g., through intensive media coverage) or particularly dramatic and salient (as is usually the case with the emergence of a novel virus). It is important to note that, depending on the amount of readily available information on the matter, the availability heuristic may lead to an overestimation of the actual probability and thus also to corresponding fears and worries.

Recently, Cohen (2020) provided evidence for the applicability of the availability heuristic in a health context. In particular, she found that exposure to celebrities suffering of COVID‐19 increased anxiety towards the disease. Likewise, Chan et al. (2018) showed that the degree of media coverage is associated with risk perceptions and protective behaviours (in this case, on the ZIKA virus). Several theoretical papers on COVID‐19 risk perceptions have made similar assumptions (e.g., Lunn et al., 2020; World Health Organization, 2020). On this basis, we argue that fears and worries about the virus itself might have been highest at the beginning of the pandemic—as the disease was rather new and represented an unknown threat which was intensively covered by the media (BBVA Research, 2020). NPIs, in turn, are well suited to reduce these feelings of virus‐related fears and worries as they reduce the number of confrontations with potentially infected other individuals. This assumption is supported by recent findings showing that fear of the virus and perceptions of its dangerousness lead to a higher compliance with NPIs (Abdelrahman, 2020; Harper et al., 2020). Furthermore, it is in line with the risk‐as‐feelings approach by Loewenstein et al. (2001), which suggests that “emotion—specifically affective responses to threats such anxiety, worry, and fear—is viewed as a dimension of risk perception that is capable of exerting its own discrete and direct influence on behavior.” (Cohen, 2020, p. 728).

Taken together, these deliberations might explain why the acceptance of NPIs was rather high at the beginning of the pandemic. However, as of November 2020, the pandemic is far from over. While the German federal and regional governments considerably eased their measures during May and June 2020 due to low infection rates (Steinmetz et al., 2020), several new infection clusters emerged throughout June and July, entailing regional restitutions of the measures. This dynamic is in line with the widely received modelling study by Kissler et al. (2020), who concluded—already in March 2020—that prolonged or intermittent physical distancing may be necessary for several years. Moreover, Lewis (2020) predicted another increase of infections following a considerable easing of the measures, as they cannot, on their own, completely eliminate the virus from the population.

It is suggested that the longer NPIs are kept in place, the stronger their economic impact will be (Sachverständigenrat zur Begutachtung der gesamtwirtschaftlichen Entwicklung [German Council of Economic Experts], 2020). In fact, such measures decrease supply and demand of non‐essential goods, thus essentially “shutting down” large parts of the economy. This has drastic consequences for local retailers, shop‐ and barkeepers, and several service providers. Consequently, economists warn that the pandemic will have (and already has) severe economic consequences, such as substantial increases in unemployment rates and a sharp recession. For example, in June 2020, the German Council of Economic Experts predicted that Germany's gross domestic product would decrease by 6.5% over the year 2020 (this was later corrected to 5.0%, but the June prognosis is more relevant for our study, which covers a period from March to July). Furthermore, the World Trade Organization (2020) estimated a year‐on‐year drop in the worldwide volume of merchandise trade of around 18.5% in the second quarter of 2020. From a psychological perspective, such developments have important consequences. In addition to individual fears and worries about falling ill with COVID‐19, individuals are subjected to another subjective threat, namely the perception that the NPIs may have severe economic (and social) consequences—both for themselves and for society overall. Hence, while we argue that worries about the virus itself will likely increase the acceptance of NPIs (see earlier), we also expect that economic worries may have opposite effects—as the following paragraphs will show.

As stated earlier, the acceptance of NPIs was rather high at the beginning of the pandemic. However, as the novelty of the disease fades, worries about the virus are likely to diminish—which is in line with the availability heuristic outlined above (Tversky & Kahneman, 1973). Moreover, the expected economic downturn can be expected to increase worries regarding economic side effects as more and more people will suffer from or worry about unemployment. We argue that over time, this subjective economic threat (and the corresponding individual worries) will become more important than the subjective health threat. This is for several reasons: first, the negative economic effects of NPIs take some time to become visible—for example, companies may first tap into their financial reserves before they start dismissing staff. Second, the economic crisis may become increasingly salient over time, for instance, as more and more yet unaffected people will learn about neighbours or friends losing their jobs and will be increasingly confronted with media coverage on the crisis. Third, considering that in Germany, the NPIs successfully reduced the rate of infections during spring and summer, the perceived dangerousness of the virus may decrease over this time period—a potential side effect of an otherwise successful containment strategy (a phenomenon known as the “prevention paradox”; Rose, 1981). As a result, we expect that the salience of the benefits of NPIs will be increasingly outweighed by the growing salience of potential negative economic consequences.

To sum up, we thus argue that the economic threat and the corresponding individual fears and worries will lead to a reduction in the acceptance of and adherence to NPIs. The recent emergence of rallies against coronavirus measures as well as the spread of COVID‐19‐related conspiracy theories (e.g., Georgiou et al., 2020; Imhoff & Lamberty, 2020) may serve as first evidence for such dynamics. It should be noted that such developments are particularly worrying considering that “seeing others ignoring guidelines by gathering in a public place may lead individuals to assume that others assign lower importance to self‐transcendence (e.g., responsibility) or conservation values (e.g., security) than they themselves do”, which, in turn, may further reduce virus‐related worries and the willingness to engage in physical distancing (Wolf et al., 2020, p. 623). Furthermore, we suggest that virus‐related health worries and economic worries interact on an individual level, that is, the degree of economic worries will relate to a decreasing acceptance of NPIs with decreasing levels of health worries. For example, an individual with high economic and low health worries is predisposed to strongly oppose NPIs since he or she is likely to perceive such measures as unnecessary, whereas an individual with high economic and high health worries might perceive the economic turmoil caused by the NPIs as an inevitable but necessary evil. Virus‐related health worries might thus buffer the negative effects of economic worries on the acceptance of NPIs. Based on these considerations, we posit the following hypotheses, which have been pre‐registered^2^ as of April 2, 2020 (see Rosman et al., 2020).

**Hypothesis 1:** Worry about the novel coronavirus will gradually decrease over time in the focused time period (March to July 2020).

**Hypothesis 2:** Worry about the economic consequences of the pandemic will gradually increase over time in the focused time period (March to July 2020).

**Hypothesis 3:** Acceptance of NPIs will gradually decrease over time in the focused time period (March to July 2020).

**Hypothesis 4:** Worry about the virus is positively related to the acceptance of NPIs00.

**Hypothesis 5:** Worry about the economic consequences of the pandemic is negatively related to the acceptance of NPIs.

**Hypothesis 6:** There is an interaction between worry about the virus and worry about its economic consequences on the acceptance of NPIs: the negative relation between worry about the economic consequences and the acceptance of NPIs (see Hypothesis 5) is stronger if worry about the virus is low (and vice versa).

**Hypothesis 7:** The interaction outlined in Hypothesis 6 will become stronger over time in the focused time period (March to July 2020).

## STUDY 1—VALIDATING THE COSMO MEASURES

### Goals

Hypotheses were tested using data from COSMO Germany (Betsch, Wieler, Habersaat, et al., 2020). As COSMO uses single indicators with unknown reliability, we conducted, inspired by a comment of one of the reviewers in the first revision stage, a validation study on the target COSMO measures (Fiske, 1982). In this study, which was not part of our preregistration, we tested measurement models consisting of the COSMO indicators and additionally generated convergent items, and then assessed the predictive effects of hypothesised validation criteria on these indicators. Due to space limitations, we restrict the development of the hypotheses regarding Study 1 to the presentation of an overall rationale.

First, we expected a relationship between virus‐related health worries and general (i.e., not COVID‐focused) trait measures on *health anxiety* and *dispositional worry*. Both constructs represent dispositions towards worrying about negative future states with a focus on getting ill (Abramowitz et al., 2007) or overall worrisome situations (Berle et al., 2011) and, thus, should predict more specific worries.

Second, we expected relationships between validation criteria and two forms of economic worries, that is, worries about becoming unemployed and worries about recession. In particular, we expected that worries about becoming unemployed should be predicted by dispositional worry as well as by the perceived *economic impairment*, that is, an overall assessment of the impact of the pandemic on the person's job or income situation. For overall worries regarding a recession, we expected an effect of dispositional worry, but not of economic impairment, as individuals who are not economically impaired might nevertheless worry about the economic consequences of the pandemic on a more generic level.

Third, we expected the following constructs to predict the acceptance versus rejection of NPIs: *health anxiety, dispositional reactance, loneliness* and *economic impairment*. The rationale is that individuals high in health anxiety should support these measures out of fear to infect themselves (Bailer et al., 2013), while persons with high reactance scores should reject the measures due to their resistance to comply with any discretion‐reducing strategy (Hong & Faedda, 1996). Furthermore, people feeling lonely should reject NPIs (particularly stay‐at‐home orders) as these reduce the chance to get in touch with other people. Finally, the degree of economic impact should lead persons to reject the measures to prevent job loss or financial harm. Table 1 presents a summary of the expected relationships.

**TABLE 1:** Expected relationships between COSMO constructs (columns) and validation criteria (rows)

	Virus‐related health worries	Economic worries: employment	Economic worries: recession	Acceptance of stay‐at‐home orders	General rejection of NPIs
Health anxiety	**+**			**+**	**−**
Worries trait	**+**	**+**	**+**		
Reactance trait				**−**	**+**
Loneliness				**−**	**+**
Economic impairment		**+**		**−**	**+**

*Note:* + = expected positive relationship; − = expected negative relationship.

### Participants and procedure

The study was conducted throughout October and November 2020. Our sample consisted of *N* = 612 individuals, which were recruited using an online panel of an ISO 26362 certified online sample provider. Sampling was quota‐based, with sex and age approximating the respective distributions in the German general population. Consequently, an equal share of men (*n* = 303) and women (*n* = 309) participated in the study. Mean age was *M* = 50.2 (*SD* = 17.3), ranging from 18 to 83 years. Education levels were *no education* (*N* = 2), *lower secondary school* (“Hauptschulabschluss”; *N* = 82), *intermediate secondary school* (“Realschulabschluss”; *N* = 219), *university entrance qualification* (“allgemeine Hochschulreife”; *N* = 133), *university degree* (*N* = 160) and *doctorate* (*N* = 14).

All study procedures were in accordance with the 1964 Helsinki Declaration and its later amendments or comparable ethical standards. Informed consent was obtained from all individual adult participants prior to study participation. No minors were included in the study.

### Measures

#### *COSMO* *measures*

We used the following COSMO single‐item measures to test our hypotheses. All items were administered in German language and have been translated for the present article. The translated items are provided in Table 2, and the items' German versions can be found in Betsch, Korn, Felgendreff, et al. (2020) as well as in the Study 1 codebook (see Rosman et al., 2021a).

**TABLE 2:** Factor loadings of the CFA and equation‐based specification tests

	Initial model (CFA)		Final model (SEM with predictors)
Factor and indicators	Standardised loading	Sargan test for indicator misspecification	Standardised loading
*Virus‐related health worries*			
The novel coronavirus to me feels … worrying/not worrying.^a^ (aw01)	0.787	NA	0.894^b^
I am worried that I could fall seriously ill with the coronavirus. (aw02)	0.913	91.45 (12)***	NA
I often think about getting infected. (aw03)	0.882	84.31 (12)***	NA
*Economic worries: employment* (Ω = .95)			
Given the current Corona situation, how worried are you about losing your job?^a^ (ewj01)	0.914	NA	0.904
Given the current Corona situation, how worried are you that your work situation will get worse? (ewj02)	0.903	13.66 (12)	0.918
Given the current Corona situation, how worried are you that there will be negative consequences for your job? (ewjs03)	0.957	16.91 (12)	0.952
*Economic worries*: *recession* (Ω = .97)			
Given the current Corona situation, how worried are you that an economic recession will occur?^a^ (ewe01)	0.878	NA	0.876
Are you concerned about the effects of Coronavirus on the economy in general? (ewe02)	0.877	10.44 (12)	0.876
Given the current Corona situation, how worried are you that the economic impact of the Corona crisis will persist for a long time to come? (ewe03)	0.876	22.95 (12)*	0.878
*Acceptance of NPIs (e.g., stay‐at‐home orders)* (Ω = .91)			
It should only be allowed to leave home for professional, health or urgent reasons.^a^ (acc01)	0.783	NA	0.783
People should be encouraged to spend as much time at home as possible to slow down the spread of the coronavirus. (acc02)	0.905	13.13 (12)	0.901
I think it is appropriate for the government to take measures to encourage people to stay at home. (acc03)	0.944	11.83 (12)	0.948
*General rejection of NPIs (α = .92)*			
I think the measures currently being taken are greatly exaggerated.^a^ (rej01)	0.912	NA	0.927
There is no justification for the current interference with individual freedom. (rej02)	0.898	29.48 (12)***	0.870
The measures only restrict personal freedom, but do not achieve much. (rej03)	0.931	36.04 (12)***	NA

*Note:* Cronbach's alpha calculated as omega requires at least three items; *α =* Cronbach's alpha, Ω = McDonald's omega.

^a^ Respective COSMO item.

^b^ Loading resulting from correction for measurement error attenuation.

***p < .001; *p < .05.

*Virus‐related health worries*. Individual worries about the virus were assessed using the item “The novel coronavirus to me feels … not worrying/worrying”, with responses recorded on a 7‐point scale ranging from 1 to 7. As can be seen in the item text, this measure directly focuses on individual worries about the virus itself.

*Economic worries: worries about becoming unemployed*. The following item was used: “Given the current Corona situation, how worried are you about losing your job?”, with responses recorded on a 7‐point scale ranging from 1 (“don't worry at all”) to 7 (“worry a lot”).

*Economic worries: worries about recession*. The following item was used: “Given the current Corona situation, how worried are you that an economic recession will occur?”, with responses recorded on the same 7‐point scale as the item on worries about becoming unemployed. In contrast to the item on virus‐related health worries, both items on economic worries more strongly focus on the consequences of the pandemic (i.e., as indicated by the notion “Corona situation”), possibly also including worries about the (economic) consequences of the mitigation measures.

*Acceptance of NPIs*. Since NPIs are subject to changes over time, we opted against building an aggregate measure of different NPI items, but instead investigated one central item which assesses the general rejection of such measures: “I think that the currently implemented measures are greatly exaggerated”. Similar to the worry items from above, responses to this item were recorded on a 7‐point scale ranging from 1 to 7 (“strongly disagree” to “strongly agree”). It is important to note that the item assesses the acceptance of NPIs at the time of data collection, and that these measures were gradually relaxed from mid‐April onwards in Germany (Steinmetz et al., 2020). This might potentially reduce the magnitude of effects with regard to Hypothesis 3. Therefore, to gain a more precise indicator of one central NPI that has been widely used, we additionally, as a secondary outcome, investigated the acceptance of stay‐at‐home orders using the item “It should only be allowed to leave one's house for professional, health, or urgent reasons”, which again was responded to on a 7‐point scale ranging from 1 to 7.

#### Additional items

For each of the five COSMO items, we generated two additional items, which we expected to reflect the same underlying common factor. This was done to enable us to conduct CFAs using latent factors, and resulted in a set of 10 additional items. These items can be found in Table 2.

#### Validation criteria

*Health anxiety*. We used three adapted items from the instrument by Bailer et al. (2013). An example is “Even before the Corona crisis, I spent a lot of time worrying about my health.” We added the reference to the Corona crisis to explicitly delineate between general and pandemic‐related health anxiety. The response format ranged from 1 (“do not agree at all”) to 7 (“fully agree”). McDonald's Omega, representing the internal consistency, was 0.86.

*Dispositional worry*. Dispositional worry was measured using three items from the scale by Berle et al. (2011). An example is “Many situations make me worry.” The response format was a 7‐point rating scale ranging from 1 (“not typical of me at all”) to 7 (“very typical of me”). Omega was 0.89.

*Dispositional reactance*. We used four items by Hong and Faedda (1996) to measure overall reactance. An example is “I become angry when my freedom of choice is restricted.” Responses were possible on a 7‐point rating scale ranging from 1 (“do not agree at all”) to 7 (“fully agree”). Omega was 0.89.

*Loneliness*. We measured loneliness with three items by Neto (2014). An example is “I feel lonely at the moment”. The response format was a 7‐point rating scale ranging from 1 (“do not agree at all”) to 7 (“fully agree”). Omega was 0.93.

*Economic impairment*. We measured economic impairment with an index of four self‐generated items. These items concern (a) whether the crisis has had an adverse impact on the economic sector in which the person works, (b) whether the person was on a furlough at least once during the crisis, (c) whether the person has lost his/her job during the crisis and (d) whether the company of the person was harmed by the crisis. Each item was binary with 0 indicating “no” and 1 indicating “yes”. As economic impairment represented a multi‐faceted index, we refrained from calculating a measure of internal consistency.

### Statistical analysis

The complete analysis code as well as the dataset and results of Study 1 can be found in (2021a). All analyses were conducted in R (R Core Team, 2020); we thereby used the packages “lavaan” (Rosseel, 2012) and MIIVsem (Fischer et al., 2020). In the first step, CFAs focused on testing the respective factor model with the goal of attaining a fitting model with a set of at least two indicators per latent variable (Antonakis & House, 2014). Subsequently, the aforementioned validation criteria were added as predictors of the five factors. The validation criteria were added as manifest composites as the goal of the procedure was to test the factor structure of our target constructs and not of the criteria, and as we wanted to avoid a misfit due to a misspecification of the criterias' measurement models. Modelling structural effects instead of investigating mere correlations (as is often done in typical validation studies) provides stronger validation evidence as correlations may be biased or even spurious based on omitted confounders or relationships with other included predictors. Instead, multivariate analyses result in unique relationships between predictors and criteria, adjusted for dependencies among the predictors. Besides this, adding criteria to a formerly fitted factor model results in creating new hurdles concerning the factor structure because the added variables result in new testable implications which the initial model did not contain. This increases the support for the validity of a model, especially if some kind of re‐specification had to occur in the first step. Finally, we estimated the effects of all predictors to avoid confounding a misfit due to falsely omitted effects with the misfit due to the misspecification of the target factor structure.

We used two forms of statistical tests to evaluate the models. The first was the model chi square test which is a summary test of all testable implications of a model (e.g., the local independence assumptions in a factor model; McIntosh, 2007; Kline, 2016). Regarding fit indices (i.e., the CFI and RMSEA), we report them for descriptive purposes only. As a second test, we used the approach by Bollen (2019) to test the specification of factor‐to‐indicator effects by means of “model‐implied instrumental variables” (MIIVs). Although the overall chi‐square test is rather uninformative about the source of the misfit, MIIVs allow identifying misspecified indicators by means of an equation‐specific test of endogeneity (i.e., the Sargan test). Finally, we inspected standardised residuals (i.e., differences between empirical and model‐implied covariances) to investigate potential local misfit. The models were estimated with full information maximum likelihood (FIML) and the Yuan–Bentler correction of the chi‐square test and standard errors (which corrects against nonnormality of the indicators).

## RESULTS

The first CFA model resulted in a misfit as the overall chi‐square test was highly significant (*χ* ^2^ = 192.18, *df* = 80, *p* < .0001, CFI = .98, RMSEA = .051). It should be noted that the fit indices (CFI and RMSEA) would have resulted in mistakenly accepting the model as a valid representation of the data. Alongside the significant chi‐square test, the single‐equation Sargan tests pointed to specific indicators as problematic. Finally, the standardised residuals pointed to informative patterns of overprediction versus underprediction of covariances between the indicators of the virus‐related health worries factor and all other variables, indicating that a common factor cannot capture the relationships among these three items. Table 2 shows the loadings of this model and associated Sargan tests.

As a modest and careful adaptation to the misfit, we eliminated the items aw02 (“I am worried that I could fall seriously ill with coronavirus”) and rej02 (“The measures only restrict personal freedom but do not achieve much.”) based on the amount of misspecification indicated by the Sargan test and a re‐consideration of the question wordings pointing to specifics not reflected in the supposed factors (“seriously ill”, “do not achieve much”). We realise that there is a discussion in the literature about whether eliminating indicators is legitimate (vs. respecificing the overall structure). However, we followed the logic by Herting and Costner (2000) as our goal was not to test and identify the structure of a given set of indicators but to test and identify indicators that validly measure our target constructs. The respecified model yielded a non‐significant chi square test (*χ* ^2^ = 60.00, *df* = 44, *p* = .054, CFI = 1.00, RMSEA = .026) and served as a baseline for the next, more rigid test.

In the next step, validation criteria were added. The additional implications (i.e., conditional independencies between criteria and COSMO indicators) again caused the model to misfit (*χ* ^2^ = 138.20, *df* = 95, *p* = .003, CFI = .99, RMSEA = .03), signifying the value of adding external variables to a respecified model to avoid false overfitting. The Sargan tests pointed to a highly significant misspecification of indicators on virus‐related health worries, and the standardised residuals supported this result by showing a symmetric overprediction versus underprediction of the correlations between both indicators and all other variables in the model. The theoretically most plausible interpretation for this is that there is no central underlying response‐generating factor and that the target COSMO item (“The novel coronavirus to me feels…worrying/not worrying.”) is more general than the added item which is more focused on the health aspects of the virus. We stress that relying on fit indices would again have resulted in keeping this set and thus, have led to a misinterpretation of the factor (and COSMO item) as “virus‐related health worries”. A final model without the added item fitted well (*χ* ^2^ = 92.28, *df* = 80, *p* = .16, CFI = 1.00, RMSEA = .016) and all Sargan tests were non‐significant. We therefore decided, for this article, to re‐label the target COSMO item formerly described as “virus‐related health worries” to “virus‐related worries”. It should be noted that is a deviation how the construct was introduced in the pre‐registration. However, a re‐labelling is more in line with our hypotheses as they explicitly address “worry about the virus”, and that it is also more in line with our item wordings (see Table 2). Instead of using the single COSMO item as an error‐prone manifest variable, we approximated its measurement error using the Cronbach's alpha estimate of the two indicators of the best fitting factor model in order to correct the indicator against attenuation. The corresponding final factor loadings are depicted in the last column of Table 2.

The estimates of the structural effects of the validation criteria can be found in Table 3. With one exception (i.e., the non‐significant effect of economic impairment on the acceptance of stay‐at‐home orders), all effects were significant and of substantial magnitude. Even more importantly, the differential effects support the discriminant validity of most of the constructs.

**TABLE 3:** Results of the structural regression model (standardised regression coefficients)

	Virus‐related worries^a^	Economic worries: employment	Economic worries: recession	Acceptance of NPIs (e.g., stay‐at‐home orders)	General rejection of NPIs
Health anxiety	.298***	−.026	−.078	.205***	−.109**
Worries trait	.167**	.212***	.216***	.101*	−.073
Reactance trait	−.324***	.022	.036	−.406***	.520***
Loneliness	−.071	.100	.058	−.174***	.170***
Economic impairment	−.066	.585***	.040	**−.051**	.145***

*Note*. Estimates printed in bold were hypothesised.

^a^ Re‐labelled from virus‐related health worries after the model testing procedure.

*p < .05; **p < .01; ***p < .001.

### Interim discussion

In Study 1, we validated COSMO's single‐indicator measures. We demonstrated that the measures exhibit a clear and empirically valid factor structure with strong factor loadings (average = .89) and high estimates of internal consistencies (see Table 2). This shows that using single indicators in the COSMO project instead of several indicators will not lead to a substantial attenuation bias. We realise that such high loadings and internal consistencies are unusual and may create the impression of an artificial redundancy among indicators. Such an impression, however, implies the data's non‐accordance to the factor model and presence of a series‐effect model (e.g., when responses of earlier items affect subsequent items, see Bollen & Medrano, 1998), as well as the emergence of meaningless and artificial common factors. In this regard, we emphasise how important a stringent test of the factor structure is and that incorporating predictors into the test of the factor structure serves to test the factor structure and to rule out such a meaningless‐factor hypothesis. According to our analyses, our fitting model and the effects of the validation criteria support both the correctness of the factor model (vs. series‐effect model) and non‐arbitrariness of the factors. It should be noted that this was the case despite having only two to three indicators for each factor. From this perspective, our study supports the philosophy to focus on a small number of key indicators instead of a large and, often consequently, heterogeneous set of items, which contradicts the essential assumptions inherent in the common factor model (Hayduk & Littvay, 2012).

Besides these strengths, Study 1 has the important limitation that we cannot rule out an upward bias of the effects of the validation criteria and COVID factors due to unobserved confounding (e.g., common method bias or evaluative response tendencies). We tried to reduce that possibility by focusing on trait measures and assume that mutually controlling the predictors also reduced the strength of confounding. We emphasise the usefulness of the MIIV approach in this regard as it can also be used for testing effects between latent variables against the danger of endogeneity (Foster & McLanahan, 1996) when all model variables are common factors. Likewise, Antonakis and House (2014) showed that simple experimental manipulations can also serve testing a factor structure and providing validity evidence. In this regard, the authors argued that specifying structural models with theoretically based effects provide stronger evidence than calculating mere correlations (i.e., the nomological net). With our analysis, we hopefully provided some support for the fruitfulness of such a perspective.

## STUDY 2—TESTING THE PRE‐REGISTERED HYPOTHESES

### Participants and procedure

Since the beginning of March 2020, the COSMO Germany data have been collected regularly (once per week until wave 13; every second week since wave 14) using non‐probability‐based sampling by the same sample provider that was already used for Study 1. The COSMO study's research design is multiple cross‐sectional, that is, for each data collection wave, a different set of participants is recruited. The online sample provider thereby ensures the independence of samples across time, meaning that a fresh set of participants is recruited for every wave and that there is no overlap in participants between waves. It should be noted that this does not correspond to a longitudinal design, in which *the same* participants would have been surveyed multiple times (i.e., at each wave). All participants are German‐speaking residents of Germany, and are matched (using quota configurations) to the German general population as captured by census data regarding age, gender, and residency in the German federal states. As the pre‐registration was finalised by April 02, 2020, data collection waves 1–3 were omitted from all analyses. Hence, only waves 4 to 16 were considered for the present study, resulting in 13 waves overall and an analysis period ranging from March 24, 2020 to July 07, 2020. The average sample size per wave was *N* (mean) = 1007, and the total sample size across all waves was *N* (overall) = 13,094. Of these 13,094 participants, 49% were female and the mean age was *M* = 46.2 (*SD* = 15.8; range: 18–87).

Again, all study procedures were in accordance with the 1964 Helsinki Declaration and its later amendments or comparable ethical standards. Study 2 was approved by the institutional review board at the University of Erfurt (#20200302/20200501). Informed consent was obtained from all individual adult participants prior to study participation. No minors were included in the study.

### Measures

All hypotheses were tested using the set of COSMO items described in the Measures section of Study 1 (i.e., virus‐related worries, worries about employment, worries about recession, acceptance of stay‐at‐home orders, general rejection of NPIs).

### Statistical analysis

The complete analysis code as well as the results of Study 2 can be found in Rosman et al. (2021a, 2021b). To facilitate the interpretation of results, items on virus‐related worries as well as on the general rejection of NPIs were inverted^3^ prior to data analysis (which is why we also renamed the NPI item to “general acceptance of NPIs”). Consequently, higher values on all worry/acceptance measures indicate higher levels of worry/acceptance. Moreover, we accounted for the hierarchical data structure (i.e., individuals clustered in measurement occasions) by employing multi‐group structural equation models with “time” (i.e., measurement occasion) as grouping variable in R (R Core Team, 2020). We thereby used the package “lavaan” (Rosseel, 2012) and its functions on model invariance testing in multi‐group models.

In a first step, we tested if mean scores on our variables differed across time (Hypotheses 1–3). To do this, we compared, by means of a likelihood ratio test, an unrestricted (and thus saturated) baseline model (i.e., allowing for variation in mean values across measurement occasions) to a restricted model which assumed that mean values were invariant across time. Apart from that, no assumptions on relationships between variables were made in the models we used in this first step (i.e., all intercorrelations between variables were freely estimated). After this likelihood ratio test, we inspected the amount of overlap in 95% confidence intervals (CIs) of mean scores to test whether the empirical pattern of change corresponded to the expected pattern of change outlined in Hypotheses 1 to 3. In order to account for multiple testing and to ensure that only significant mean differences are interpreted, we additionally calculated Turkey‐corrected post hoc tests. These can be found in the R Markdown file for Study 2 (see Rosman et al., 2021a, 2021b).

In a second step, we examined relationships between variables separately for each of the 13 measurement occasions (Hypotheses 4–6) and how these relationships changed across measurement occasions (Hypothesis 7). To investigate the relationships between virus‐related worries, economic worries, and acceptance (Hypotheses 4–6), we predicted acceptance measures by worry variables in our multi‐group model. To allow for meaningful comparisons of these regression coefficients across measurement occasions, we centred each variable on the corresponding measurement occasion mean (cf. centring within cluster; Enders & Tofighi, 2007)—thereby accounting for differences in measurement‐occasion‐specific mean values. Moreover, to further facilitate the interpretation of the estimated regression coefficients, we also divided each variable by its measurement occasion‐specific standard deviation, thus obtaining standardised regression coefficients. Finally, to test Hypotheses 6 and 7, we added interaction terms to the multi‐group model by multiplying these standardised variables. In line with our procedure for testing Hypotheses 1 to 3, we first tested if regression coefficients were invariant over time and decided, based on this test, whether to inspect measurement occasion‐specific regression coefficients or time‐invariant regression coefficients to investigate Hypotheses 4 to 6. Again in line with our procedures for the first step, Hypotheses 4 to 7 were subsequently tested based on 95% CIs^4^ of standardised regression coefficients. More precisely, we examined whether regression coefficients significantly differed from zero for Hypotheses 4 to 6 (i.e., *if* there was a significant positive or negative effect), and, regarding Hypothesis 7, we inspected the amount of overlap in the CIs of the interaction effects.

One multi‐group structural equation model was estimated for each step (i.e., one for Hypotheses 1–3 and one for Hypotheses 4–7). All relationships were thus analysed simultaneously and all worry measures, interaction variables and NPIs were included in the same model. Therefore, our regression coefficients can be interpreted as *partial* regression coefficients (in line with “ordinary” regression coefficients in multiple regression models). Additionally, to obtain a target model that was as parsimonious as possible, we performed, for each of the two models, model invariance tests to determine if (co)variances significantly differed across measurement occasions. Finally, as specified in our pre‐registration, incomplete cases (4.47%) were treated as “missing” in the multi‐group model.

### Results

In our analyses on changes over time (i.e., the first step described in the *Statistical Analysis* section), likelihood ratio tests showed that mean values significantly differed between measurement occasions (Δ*χ* ^2^ = 1641.50, *df* = 60, *p* < .001), and that restricting variances (Δχ^2^ = 129.98, *df* = 60, *p* < .001) or covariances (Δ*χ* ^2^ = 324.92, *df* = 120, *p* < .001) also significantly impaired model fit. Thus, we inspected mean value estimates of a completely unrestricted model to test Hypotheses 1 to 3 (i.e., this model is a fully saturated model which is why no fit statistics are reported here). The corresponding model‐implied 95% CIs of mean values are given in Table 4.

**TABLE 4:** Descriptive statistics and 95% confidence intervals of study variables

Variable		Virus‐related worries			Economic worries: employment			Economic worries: recession			General acceptance of NPIs			Acceptance of stay‐at‐home orders		
Date	N	M	SD	CI	M	SD	CI	M	SD	CI	M	SD	CI	M	SD	CI
24.03.2020	1026	5.302	1.506	5.210, 5.394	2.821	2.069	2.694, 2.947	5.390	1.482	5.299, 5.481	5.622	1.732	5.516, 5.728	5.082	1.917	4.965, 5.199
31.03.2020	988	5.302	1.578	5.203, 5.400	2.926	2.061	2.798, 3.055	5.403	1.476	5.311, 5.495	5.465	1.792	5.353, 5.576	4.819	1.945	4.698, 4.940
07.04.2020	987	4.961	1.665	4.858, 5.065	2.874	2.038	2.747, 3.001	5.348	1.516	5.253, 5.442	5.198	1.899	5.079, 5.316	4.349	2.023	4.222, 4.475
14.04.2020	998	4.873	1.616	4.773, 4.973	2.745	2.044	2.619, 2.872	5.265	1.512	5.171, 5.358	5.215	1.913	5.097, 5.334	4.202	2.033	4.076, 4.329
21.04.2020	963	4.774	1.658	4.669, 4.878	2.671	1.904	2.551, 2.791	5.178	1.444	5.086, 5.269	5.211	1.879	5.092, 5.329	3.979	2.013	3.852, 4.106
28.04.2020	986	4.801	1.667	4.697, 4.905	2.772	1.987	2.648, 2.896	5.242	1.518	5.148, 5.337	4.820	2.004	4.695, 4.946	3.700	2.094	3.569, 3.831
05.05.2020	963	4.542	1.613	4.440, 4.644	2.717	1.987	2.591, 2.842	5.115	1.587	5.015, 5.215	4.687	2.002	4.561, 4.814	3.395	2.004	3.268, 3.521
12.05.2020	983	4.556	1.670	4.452, 4.661	2.742	2.032	2.615, 2.869	5.140	1.553	5.043, 5.237	4.800	2.042	4.672, 4.927	3.318	1.990	3.194, 3.443
19.05.2020	922	4.502	1.717	4.391, 4.613	2.785	1.999	2.656, 2.914	5.156	1.517	5.058, 5.254	4.684	1.993	4.556, 4.813	3.371	1.985	3.243, 3.499
26.05.2020	881	4.490	1.681	4.379, 4.601	2.866	2.003	2.734, 2.998	5.163	1.499	5.064, 5.262	4.841	1.946	4.713, 4.970	3.058	1.903	2.932, 3.184
09.06.2020	918	4.634	1.658	4.527, 4.741	2.876	1.980	2.748, 3.004	5.146	1.513	5.048, 5.244	4.804	1.958	4.677, 4.931	3.197	1.998	3.068, 3.326
23.06.2020	946	4.442	1.687	4.334, 4.549	2.823	2.065	2.692, 2.955	5.053	1.589	4.952, 5.154	4.742	2.024	4.613, 4.871	3.060	1.965	2.935, 3.185
07.07.2020	948	4.497	1.748	4.386, 4.608	2.601	2.028	2.472, 2.730	5.004	1.617	4.901, 5.107	5.018	1.969	4.893, 5.143	2.746	1.893	2.625, 2.866

*Note. N* = sample size (for each wave); *M* = mean; *SD* = standard deviation; CI = 95% confidence intervals; see https://projekte.uni‐erfurt.de/cosmo2020/web/explorer/ for a graphical overview of these data.

As can be seen in Table 4, a consistent decrease in virus‐related worries occurred in March and April—but not in May, June and July (there was only one overlap in CIs [on June 09]). Hence, the decline in virus‐related worries seemed to have largely stopped by May. Thus, Hypothesis 1 is partially supported.

Contrary to our expectations, we did not observe any increases in worries about the economic consequences of the virus (see Table 4). Surprisingly, worries about a recession—while in general rather high—even seemed to decrease a bit. This is especially true for the two most recent measurement occasions (June 23 and July 7) compared to the first three (March 24 to April 07). Furthermore, regarding worries about unemployment, the pattern of change was rather unsystematic and clearly not pointing towards any significant increase. Consequently, Hypothesis 2 is fully rejected.

In Hypothesis 3, we expected the acceptance of NPIs to gradually decrease over time. This was clearly supported by our data on the acceptance of stay‐at‐home orders, which strongly decreased from March to May and still seemed to be decreasing—albeit to a smaller extent—in June and July (see Table 4). With regard to the general acceptance of NPIs, we found a corresponding decrease in March and April, but the bottom of this trajectory seemed to have been reached in May and June. In fact, the observed value for general acceptance of NPIs on July 7 was the highest observed value since April 21. Taken together, both findings imply the partial confirmation of Hypothesis 3.

For the standardised model used to test Hypotheses 4 to 7, a likelihood ratio test showed that restricting covariances to be time‐invariant significantly impaired model fit (Δ*χ* ^2^ = 344.83, *df* = 132, *p* < .001). Consequently, this restriction was discarded. Regarding our hypotheses on the relationships between worry and NPI acceptance, the corresponding likelihood ratio test implied that regression coefficients differed significantly between measurement occasions (Δ*χ* ^2^ = 253.35, *df* = 120, *p* < .001). In other words, this model imposed no ‘real’ restriction on the data (i.e., we only ‘restricted’ mean values and variances of standardised variables to be equal), which is why this model can be considered as a fully saturated model, too. Therefore, Hypotheses 4 to 7 were subsequently tested based on the measurement occasion‐specific regression coefficients given in Tables 5 and 6. More specifically, for each worry variable, 26 regression coefficients (effects across 13 measurement occasions on two NPI variables) were inspected based on their CIs.

**TABLE 5:** Standardised effects on the acceptance of stay‐at‐home orders and 95% confidence intervals

	Virus‐related worries		Economic worries: employment		Economic worries: recession		Virus‐related worries * worry about recession (interaction)		Virus‐related worries * worry about becoming unemployed (interaction)		
Date	EST	CI	EST	CI	EST	CI	EST	CI	EST	CI	R^2^
24.03.2020	0.213	0.152, 0.274	−0.085	−0.145, −0.024	0.087	0.024, 0.149	0.056	0.002, 0.109	0.041	−0.017, 0.098	0.065
31.03.2020	0.290	0.228, 0.351	0.008	−0.054, 0.069	0.015	−0.048, 0.078	0.079	0.024, 0.133	−0.026	−0.085, 0.033	0.096
07.04.2020	0.305	0.244, 0.366	0.022	−0.039, 0.084	−0.056	−0.119, 0.007	0.016	−0.039, 0.071	0.006	−0.053, 0.065	0.090
14.04.2020	0.234	0.173, 0.294	0.121	0.060, 0.182	−0.116	−0.178, −0.053	0.082	0.028, 0.136	0.046	−0.012, 0.105	0.085
21.04.2020	0.209	0.148, 0.271	0.006	−0.055, 0.067	−0.040	−0.102, 0.021	0.050	−0.004, 0.104	−0.017	−0.075, 0.042	0.050
28.04.2020	0.207	0.147, 0.267	0.058	−0.003, 0.119	−0.102	−0.164, −0.040	0.008	−0.046, 0.062	−0.013	−0.072, 0.045	0.051
05.05.2020	0.262	0.201, 0.324	0.100	0.038, 0.162	−0.068	−0.131, −0.004	0.051	−0.004, 0.107	−0.039	−0.099, 0.021	0.079
12.05.2020	0.264	0.203, 0.325	0.107	0.046, 0.168	−0.086	−0.149, −0.023	−0.011	−0.066, 0.044	−0.045	−0.105, 0.014	0.081
19.05.2020	0.319	0.256, 0.382	0.097	0.033, 0.161	−0.091	−0.155, −0.026	0.034	−0.023, 0.090	−0.010	−0.072, 0.052	0.110
26.05.2020	0.215	0.150, 0.279	0.117	0.053, 0.182	−0.057	−0.123, 0.010	0.017	−0.041, 0.076	−0.055	−0.118, 0.008	0.063
09.06.2020	0.229	0.166, 0.292	0.117	0.053, 0.181	−0.044	−0.107, 0.020	0.060	0.003, 0.116	−0.002	−0.064, 0.060	0.075
23.06.2020	0.175	0.113, 0.237	0.112	0.049, 0.176	−0.125	−0.188, −0.061	0.066	0.010, 0.121	−0.093	−0.154, −0.033	0.068
07.07.2020	0.220	0.158, 0.282	0.108	0.046, 0.171	−0.072	−0.136, −0.009	0.051	−0.005, 0.107	−0.062	−0.122, −0.002	0.072

*Note:* CI = 95% confidence intervals; EST = estimated effect (standardised); dependent variable = acceptance of stay‐at‐home orders.

**TABLE 6:** Standardised effects on the acceptance of NPIs in general and 95% confidence intervals

	Virus‐related worries		Economic worries: employment		Economic worries: recession		Virus‐related worries * worry about recession (interaction)		Virus‐related worries * worry about employment (interaction)		
Date	EST	CI	EST	CI	EST	CI	EST	CI	EST	CI	R^2^
24.03.2020	0.425	0.369, 0.481	−0.211	−0.266, −0.156	0.035	−0.023, 0.092	0.075	0.026, 0.124	0.061	0.008, 0.114	0.220
31.03.2020	0.437	0.381, 0.493	−0.149	−0.205, −0.092	−0.029	−0.087, 0.029	0.086	0.036, 0.136	0.051	−0.003, 0.105	0.228
07.04.2020	0.414	0.358, 0.47	−0.151	−0.207, −0.094	−0.056	−0.114, 0.002	0.015	−0.035, 0.065	0.026	−0.029, 0.080	0.203
14.04.2020	0.425	0.369, 0.481	−0.224	−0.28, −0.167	−0.071	−0.129, −0.014	0.095	0.046, 0.144	0.003	−0.051, 0.056	0.227
21.04.2020	0.447	0.390, 0.503	−0.140	−0.197, −0.084	−0.022	−0.078, 0.035	0.057	0.007, 0.106	−0.004	−0.058, 0.050	0.229
28.04.2020	0.440	0.384, 0.495	−0.187	−0.242, −0.131	−0.078	−0.135, −0.022	−0.022	−0.071, 0.027	0.046	−0.008, 0.099	0.228
05.05.2020	0.446	0.389, 0.502	−0.114	−0.171, −0.057	−0.092	−0.151, −0.034	0.016	−0.035, 0.067	−0.024	−0.079, 0.031	0.218
12.05.2020	0.423	0.367, 0.479	−0.202	−0.259, −0.146	−0.088	−0.145, −0.030	0.008	−0.043, 0.058	−0.050	−0.105, 0.005	0.220
19.05.2020	0.462	0.405, 0.520	−0.226	−0.284, −0.167	−0.040	−0.099, 0.019	−0.013	−0.064, 0.039	−0.087	−0.143, −0.030	0.257
26.05.2020	0.498	0.438, 0.557	−0.144	−0.204, −0.085	−0.086	−0.147, −0.024	−0.004	−0.058, 0.050	0.000	−0.058, 0.058	0.250
09.06.2020	0.418	0.36, 0.475	−0.129	−0.187, −0.070	−0.016	−0.074, 0.042	−0.042	−0.093, 0.010	−0.043	−0.099, 0.014	0.196
23.06.2020	0.442	0.385, 0.499	−0.147	−0.205, −0.089	−0.087	−0.145, −0.028	0.014	−0.037, 0.065	−0.080	−0.135, −0.024	0.227
07.07.2020	0.445	0.388, 0.502	−0.147	−0.205, −0.090	−0.112	−0.170, −0.054	0.028	−0.023, 0.079	−0.028	−0.083, 0.027	0.229

*Note:* CI = 95% confidence intervals; EST = estimated effect (standardised); dependent variable = general acceptance of NPIs.

The inspection of standardised regression coefficients revealed that worries about the novel coronavirus consistently had significant positive effects on both types of acceptance measures (general acceptance and acceptance of stay‐at‐home orders; all standardised effect estimates significant and ranging from 0.175 to 0.498; see Tables 5 and 6). Hypothesis 4 is fully supported.

However, results regarding Hypothesis 5 were more ambiguous. Although worries about employment had consistent negative effects on the general acceptance of NPIs (with all standardised effect estimates significant and ranging from −0.114 to −0.226, see Table 6), this was not true for the acceptance of stay‐at‐home orders. Instead, on this measure, significant positive effects were observed in 8 out of 13 measurement occasions (see Table 5). For worries about recession, most (23 out of 26) regression coefficient estimates were negative (see Tables 5 and 6)—however, only 14 of these estimates reached statistical significance, and even those that did so were generally very small (with only four of them larger than 0.100). Thus, we deem Hypothesis 5 to be only partially confirmed.

Finally, as can be seen in Tables 5 and 6, only 14 (out of 52) interaction effects between virus‐related and economic worries were significant, with 10 indicating a positive interaction between worry variables and 4 pointing towards a negative interaction.^5^ Moreover, no consistent significant changes in their magnitude over time were found, which is why we conclude that neither Hypothesis 6 nor Hypothesis 7 is supported by our data.

## OVERALL DISCUSSION

The present study investigated the interplay between virus‐related worries and economic worries on the acceptance of NPIs during the beginning of the SARS‐COV‐2 outbreak in Germany. To do so, we re‐analysed data from the COSMO Germany survey (Betsch, Wieler, Habersaat, et al., 2020), a recurring monitoring survey on psychological and behavioural aspects associated with the pandemic.

### Main findings

Supporting our expectations, results showed that virus‐related worries gradually decreased from March to April 2020. This finding is in line with established psychological theories on judgement heuristics (e.g., the availability heuristic; Tversky & Kahneman, 1973). In fact, as the novelty of the disease faded over April and May 2020, the dangers of COVID‐19 might no longer have come to mind as easily—for example because of reduced media coverage on the virus itself and increasing coverage on the easing of measures. This would, obviously, be associated with a decrease in virus‐related worries. It should, however, be noted that we did not directly test Tversky and Kahneman's (1973) assumptions, but that we merely applied them to individual reactions to COVID‐19. Supporting our theoretical claims, evidence on the availability heuristic's role in the context of COVID‐19 risk perceptions is nevertheless growing. For example, in a series of experimental and observational studies, Abel et al. (2020) showed that the availability heuristic contributed to biased beliefs on COVID‐19 risk perceptions. Among others, experimentally inducing cognitive load, which has long been used to trigger availability heuristics, led to increased risk perceptions, and, furthermore, knowing of a person that had died from the virus was also associated with an overestimation of risk perceptions (Abel et al., 2020).

Nevertheless, alternative explanations for our results that do not relate to availability heuristics are possible. For example, over the study period, it became increasingly clear that at‐risk groups (e.g., individuals with pre‐existing conditions and higher age) are more prone to severe COVID‐19 illness, whereas young and healthy persons often experience rather mild symptoms (e.g., Zhou et al., 2020). Considering that a large proportion of our sample did *not* belong to at‐risk groups, our findings on decreasing virus‐related worries might thus not only reflect decreased novelty, but might also be related to growing knowledge on the disease itself. In contrast to our expectations, however, the decrease in virus‐related worries ended at the beginning of May 2020, resulting in a rather constant level of virus‐related worries from May to July. Psychological phenomena such as the availability heuristic (e.g., Tversky & Kahneman, 1973) might thus have played a stronger role in the beginning of the pandemic, and virus‐related worries might correspondingly have diminished earlier than expected. However, it should also be considered that in other studies, psychological distress associated with COVID‐19 decreased at least until June 2020. For example, using a German convenience sample, a longitudinal study by Bendau et al. (2020) found consistent decreases in specific COVID‐19‐related anxiety from April to June 2020. Moreover, in the UK Household Longitudinal Study (Daly et al., 2020), mental health problems decreased from April to June 2020, too (although it is important to note that this study's survey items, such as “lost much sleep over worry” (p. 3) do not delineate between virus‐related and economy‐related worries). In sum, this clearly indicates a need for more research on how phenomena such as the availability heuristic evolve over time, and whether other factors (e.g., information‐seeking behaviour) might also play a role in individual pandemic‐related worries.

With regard to Hypothesis 3, we observed a linear and rather steep decline in the acceptance of stay‐at‐home orders over the entire observation period, and found that an increasingly large proportion of participants indicated that the current measures were exaggerated—at least until the end of April 2020. This could be explained by the easing of NPIs from mid‐April onwards (Steinmetz et al., 2020), and indicates that our data on the measures being exaggerated underestimate the decrease in the acceptance of NPIs over time—a potential explanation for the effects fading out beginning with May 2020. Nevertheless, these findings show that the acceptance of NPIs diminished over time (and with decreasing cases), which confirms the results of a German scenario‐based study by Gollwitzer et al. (2020), who found that lockdown length negatively affected the acceptance of such rather restrictive measures. These findings further underline the importance of investigating potential predictors of the acceptance of NPIs—as we did in Hypotheses 4 to 7.

Regarding Hypothesis 4, our data revealed significant effects of virus‐related worries on the acceptance of NPIs. More specifically, individuals with higher virus‐related worries reported more acceptance of NPIs and vice versa, which is again in line with current research on COVID‐19 (e.g., Harper et al., 2020). Of note is that the corresponding effects were stronger for the more general item on the measures being exaggerated compared to the acceptance of stay‐at‐home orders. This may be because individuals had realised that stay‐at‐home orders were only one way to deal with the pandemic, and that alternative measures such as the compulsory use of masks may also be effective. Moreover—even though we concede that this interpretation is somewhat speculative—it may indicate that trust in how the government generally deals with the pandemic depends, among others, on virus‐related worries.

An unexpected result, however, was the consistency of the level of economic worries across time, with worries about becoming unemployed remaining rather constant, and worries about a recession even slightly decreasing over the last two waves. This may be because of the vigorous government interventions in Germany, such as furloughs, unlimited loans to companies, and changes in bankruptcy legislation. These interventions, which were received very positively in Germany, might have achieved two goals: alleviating the economic and societal impact of the pandemic, and preventing an increase in individual economic worries. This might also explain why the relationship between economic worries and the acceptance of NPIs was less consistent than expected, and why we found no interactions between virus‐related and economic worries on the acceptance of NPIs. In fact, we had initially expected that high economic worries combined with low virus‐related worries would lead to maximum rejection of NPIs as individuals consider the imposed measures to be unnecessary. However, individuals with high economic worries who nevertheless see that the government is doing quite well in alleviating the negative consequences of NPIs might well conceive the government's actions as more reasonable overall, thereby also reducing their views of NPIs as unnecessary. This, in turn, might lead to a situation where virus‐related worries and economic worries are rather independent in their effects on the acceptance of NPIs, thus explaining the absence of corresponding interactions.

### Limitations and future directions

Another explanation for the unexpected results on economic worries is directly related to their measurement. In fact, worrying about unemployment strongly depends on the economy sector in question, and individuals who had already become unemployed may have had trouble responding to the corresponding item (even though the item includes an “if applicable” category). Moreover, Germany has been experiencing a recession for some time now, which is why asking participants if they worry that a recession *might* occur is problematic, too (but consider that this item performed fine in our validation study). In addition to these limitations regarding our measurement of economic worries, one might also, on a more general level, question the reliability and validity of our measurement approach, which solely draws on single items. Although our validation study provides ample support for these items' construct validity, single items are often limited in their ability to fully capture the breadth and depth of the constructs in question. For example, our items do not allow us to disentangle different types of virus‐related worries (e.g., worries about psychological vs. physical damage), which is why the generalisability of our conclusions is reduced.

Moreover, the serial cross‐sectional nature of our design is far from perfect. In fact, serial cross‐sectional studies do not allow to investigate the effects of person‐level covariates on changes in the constructs in question, which is why our study might be more descriptive than explanatory. Furthermore, a longitudinal study, compared to one employing a serial cross‐sectional design, would have required fewer participants to gain the same level of robustness of findings. What speaks in favour of our study, however, is its large sample size per wave and the comparability of samples across waves (due to quota‐based recruiting), which makes it unlikely that the pattern of mean differences between waves we observed for H1 to H3 came about through chance. In this regard, it should also be pointed out that measuring our study variables thirteen times in the same participants, as would be required in a longitudinal design, bears some methodological challenges, too (e.g., respondent fatigue).

Furthermore, the generalisability of our findings to an international context remains an open question. For example, the effects of virus‐related worries on the acceptance of NPIs might be moderated by governmental communication strategies. In fact, according to social cognitive models (e.g., Rogers, 1975), health‐related intentions are not only shaped by fear appeals and risk perceptions, but also by beliefs in the efficacy of a coping response (Glöckner et al., 2020). Hence, if controlling the spread of the pandemic is portrayed as an unrealistic goal (as was recently done by the White House; Tanne, 2020), virus‐related worries might not have the same effects as if governmental communication was more positive about the effects of NPIs. Furthermore, changes in attitudes towards NPIs as well as in individual worries obviously depend on how the country in question deals with the pandemic and its economic consequences. For example, using topic modelling techniques on Twitter data, Doogan et al. (2020) showed that countries that react early and invest in clear and empathic communication about the pandemic and its consequences, maintained higher adherence to NPIs. Denmark's reaction to the first wave of the pandemic can be seen as a role model in this regard (Olagnier & Mogensen, 2020). Correspondingly, in the COSMO Denmark survey (Böhm et al., 2020), no evidence for so‐called pandemic fatigue was found, with virus‐related worries increasing from March to June 2020, and economic worries decreasing. It should be noted that this pattern is the opposite of what we had expected (and partially confirmed with regard to Germany). While interpreting such differences between countries is subject to a multitude of methodological fallacies (e.g., correlation vs. causation), the conclusion that the effects in Denmark have to do something with governmental communication strategies should at least be considered.

In addition, future research should strive for a more fine‐grained operationalisation of the constructs analysed in this article. For example, one may analyse differences between micro‐level worries (i.e., concerns about oneself or one's close others) and macro‐level worries (i.e., worries about society in general) as predictors of NPIs (Wolf et al., 2020), or investigate the effects of shared human values (e.g., self‐transcendence values such as responsibility and conservation values such as the desire for security; Schwartz, 1992) as individual predictors of virus‐related and economic worries. Analysing a different set of dependent variables, such as the willingness to support others who are affected by the pandemic, may also prove worthwhile (Wolf et al., 2020). Finally, another potential endeavour for future research would be the analysis of constructs associated with the acceptance of NPIs, such as beliefs about the measures' impact on (a) the spread of the virus, (b) the economy and (c) on society as a whole. In fact, it is theoretically plausible that the relationships between economic worries and the acceptance of NPIs might be largely fuelled by how individuals perceive the measures' effects and side‐effects. For example, the (negative) relationship between economic worries and the acceptance of the measures might be stronger for individuals emphasising the economy‐damaging effects of the NPIs, whereas the corresponding relationship might be lower for those who view the measures as an important means to control the pandemic and to eventually limit its impact on the economy. This might even explain our inconsistent results on the relationship between economic worries and the acceptance of NPIs. Such analyses would be especially fruitful when directed at specific NPIs, and we would expect that restrictive measures (e.g., stay‐at‐home orders)—compared to less strict interventions such as compulsory masks—might bear more potential for a reduced acceptance due to worries about collateral damage.

## CONCLUSIONS

In a democratic state, behavioural infection control measures only work to the extent that people adhere to them, mostly on a voluntary basis. Our study illustrates that individual differences in the perception of the pandemic and its consequences play an important role in the German public's acceptance of NPIs. Since these perceptions are shaped by the corresponding media coverage and governmental communication strategies, we see it as imperative that research results on the pandemic are disseminated to a wider audience. This particularly applies to well‐established findings such as those on the efficacy of wearing face masks or to modelling studies that illustrate how far the virus would spread without governmental interventions. Since the beginning of the pandemic, several virologists and epidemiologists have done so in an exemplary manner, and we sincerely hope that they continue with these efforts for the duration of this global health crisis.

### Conflicts of Interest

The authors declare they have no conflict of interest.

## Endnotes

^2^
It should be noted that the analyses in this article focus on the period from March to July 2020, whereas no specific time period is specified in the pre‐registration. Moreover, we replaced the notion “social distancing measures” by the (now) more common “NPI” term in Hypotheses 3 to 6.

^3^
It should be noted that this was only done in Study 2, not in Study 1.

^4^
Despite the fact that we specified one‐sided hypotheses, 95% instead of 90% CIs were used for testing our hypotheses to correct for multiple testing when having two outcome variables.

^5^
As this inconsistent pattern clearly does not support Hypothesis 6, we decided not to report simple slope tests here for the sake of readability. However, interested readers might find these analyses in the R Markdown file (see Rosman et al., 2021b).
